# Elevated circulating cell-free mitochondrial DNA level in cerebrospinal fluid of narcolepsy type 1

**DOI:** 10.1093/braincomms/fcaf125

**Published:** 2025-04-17

**Authors:** Monica Moresco, Concetta Valentina Tropeano, Martina Romagnoli, Giulia Neccia, Alessandro Rapone, Fabio Pizza, Stefano Vandi, Emmanuel Mignot, Alessandra Maresca, Valerio Carelli, Giuseppe Plazzi

**Affiliations:** Programma di Neurogenetica, IRCCS Istituto delle Scienze Neurologiche di Bologna, Bologna 40139, Italy; Programma di Neurogenetica, IRCCS Istituto delle Scienze Neurologiche di Bologna, Bologna 40139, Italy; Programma di Neurogenetica, IRCCS Istituto delle Scienze Neurologiche di Bologna, Bologna 40139, Italy; UOC Clinica Neurologica, IRCCS Istituto delle Scienze Neurologiche di Bologna, Bologna 40139, Italy; Department of Biomedical and Neuromotor Sciences, University of Bologna, Bologna 40126, Italy; UOC Clinica Neurologica, IRCCS Istituto delle Scienze Neurologiche di Bologna, Bologna 40139, Italy; Department of Biomedical and Neuromotor Sciences, University of Bologna, Bologna 40126, Italy; UOC Clinica Neurologica, IRCCS Istituto delle Scienze Neurologiche di Bologna, Bologna 40139, Italy; Department of Biomedical and Neuromotor Sciences, University of Bologna, Bologna 40126, Italy; Stanford Center for Sleep Sciences and Medicine, Stanford University School of Medicine, Stanford, CA 94305, USA; Programma di Neurogenetica, IRCCS Istituto delle Scienze Neurologiche di Bologna, Bologna 40139, Italy; Programma di Neurogenetica, IRCCS Istituto delle Scienze Neurologiche di Bologna, Bologna 40139, Italy; Department of Biomedical and Neuromotor Sciences, University of Bologna, Bologna 40126, Italy; UOC Clinica Neurologica, IRCCS Istituto delle Scienze Neurologiche di Bologna, Bologna 40139, Italy; Department of Biomedical, Metabolic and Neural Sciences, University of Modena and Reggio Emilia, Modena 42121, Italy

**Keywords:** narcolepsy type 1, cerebrospinal fluid, circulating cell-free mitochondrial DNA, cytokines, hypocretin 1

## Abstract

Narcolepsy type 1 (NT1) is a rare neurological disorder characterized by excessive daytime sleepiness and cataplexy, thought to result from an autoimmune process targeting the hypothalamic hypocretin-producing neurons. Aiming to add clues to the latter hypothesis, we investigated circulating cell-free mitochondrial DNA (ccf-mtDNA) levels in cerebrospinal fluid (CSF), a possible biomarker for neurodegeneration, neuroinflammation or immune activation, from 46 NT1 patients with low CSF hypocretin-1, compared with 32 controls. We found significantly increased ccf-mtDNA levels in NT1 patients compared with controls, which negatively correlated with CSF hypocretin-1 concentrations. Additionally, higher ccf-mtDNA levels were observed in patients with elevated number of sleep onset rapid eye movement periods. These observations imply that increased levels of ccf-mtDNA associate with reduced CSF hypocretin-1 concentrations leading to greater alteration in sleep architecture. Furthermore, cytokine profiling in CSF revealed significant changes in interleukins 6 and 18 in NT1 patients, suggesting an active neuroinflammatory process possibly linked to ccf-mtDNA release, thus pointing to a specific inflammatory signature in NT1. These findings hint a potential mitochondrial dysfunction and neuroinflammation in NT1. Further studies are needed to elucidate the underlying mechanisms and how this may reflect on therapy.

## Teaser

Moresco *et al*. report that circulating cell-free mitochondrial DNA levels are significantly elevated in the cerebrospinal fluid of narcolepsy type 1 patients compared with controls. These levels negatively correlate with cerebrospinal fluid hypocretin-1 concentrations and are associated with sleep architecture alterations. Cytokine profiling suggests a neuroinflammatory process, implying a potential mitochondrial involvement.

## Graphical Abstract

**Graphical Abstract:**
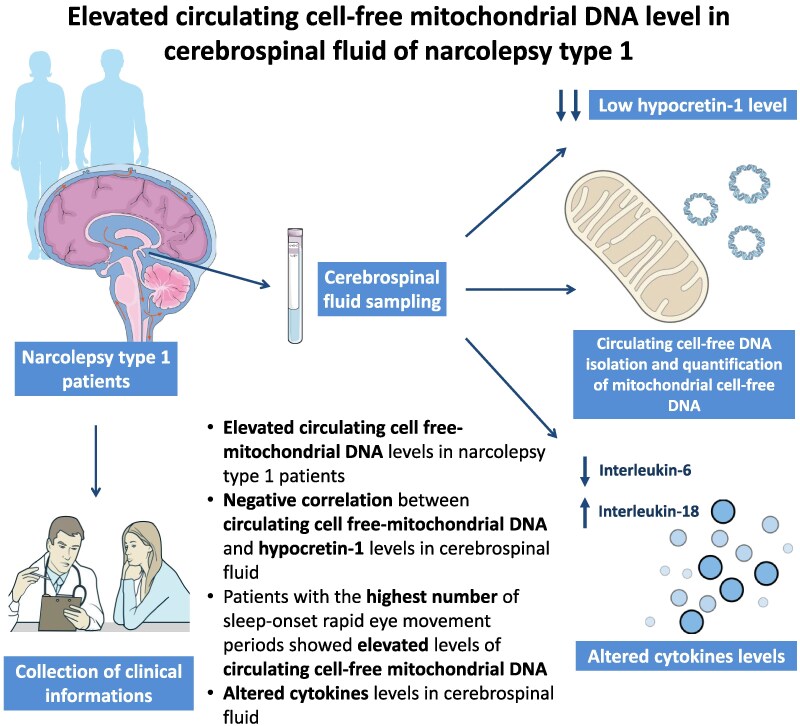

## Introduction

Narcolepsy type 1 (NT1), is a rare, lifelong central nervous system (CNS) disorder characterized by hypersomnolence, cataplexy (a sudden loss of muscle tone triggered by strong emotions), disrupted nocturnal sleep, sleep paralysis and hallucinations.^1^ Polysomnography reveals rapid sleep onset and abnormal, shortened latencies for rapid eye movement (REM) sleep. The estimated prevalence of NT1 is between 0.2 and 0.5 in 1000 individuals in Europe and North America.^1^

NT1 pathophysiology is linked to the loss of hypocretin (HCRT-1 or orexin-A) synthesizing neurons in the lateral hypothalamus. This loss is highly selective and reflected by low (<110 pg/mL) or absent cerebrospinal fluid (CSF) HCRT-1 levels, the unique biomarker for this disease.^1^ HCRT-1 is a wakefulness-associated neurotransmitter that is also secreted into CSF. In addition to CNS, this neuropeptide plays a role in peripheral organs, regulating appetite, feeding, gastrointestinal motility, energy balance, metabolism, blood pressure, neuroendocrine functions and reproductive processes.^2^ The *HCRT* gene encodes for the neuropeptide precursor prepro-hypocretin,^1,2^ from which two mature peptides, HCRT-1 and HCRT-2, are derived through proteolytic cleavage. Additionally, these neuropeptides play a role in neuroprotection by inhibiting oxidative stress, reducing apoptosis, and modulating the inflammatory response through their receptors.^3^

Although the aetiology of NT1 remains poorly understood, an immune-mediated mechanism has long been suspected due to the strong association with the MHC class II allele HLA-DQB1*06:02,^1,2^ and genetic linkage to polymorphisms in T cell receptor genes and other immune-related loci involved in antigen processing, presentation and recognition by T lymphocytes.^1,2^ Even if auto-reactive T cells targeting various antigens presented by hypocretin neurons have been extensively reported,^1^ direct evidence of hypocretin-secreting neuronal cells death, lateral hypothalamic inflammation or T cells infiltration is lacking.^1^ Supporting the immune-mediated hypothesis, an increased incidence of NT1 has been observed following H1N1 influenza infections and during pandemic vaccination campaigns with Pandemrix in the paediatric population.^1,2^ Additionally, streptococcal infections may represent another significant environmental trigger for NT1, as elevated anti-streptococcal antibodies have been found in recently diagnosed patients.^1,2^

Mitochondria play a crucial role in various cellular functions, including energy production, generation and regulation of reactive oxygen species, maintenance of calcium homeostasis, and regulation of apoptosis, particularly in high-energy-consuming tissues such as brain and muscle.^4^ Circulating cell-free mitochondrial DNA (ccf-mtDNA) consists of fragmented or whole mtDNA molecules released from cells due to mitochondrial dysfunction or cell injury.^5^ Recently, ccf-mtDNA has attracted attention as a biomarker for a range of diseases, including mitochondrial disorders,^6^ neurodegenerative diseases and other neurological conditions.^7^ Numerous studies have highlighted the relationship between mitochondrial dysfunction and increased inflammation or immune activation.^5^

We here aimed at investigating, for the first time, the CSF levels of ccf-mtDNA in NT1 patients, exploring the possible involvement of mitochondria in this rare neurological sleep disorder. Moreover, we assessed the relations between ccf-mtDNA and CSF HCRT-1 levels in CSF, as well as ccf-mtDNA and NT1 clinical features.

## Materials and methods

### Patient and control groups

The NT1 patients (n = 46) were diagnosed according to current criteria,^8^ with all participants exhibiting low (<110 pg/mL) or undetectable levels of HCRT-1 in their CSF. Patients were categorized as recent onset NT1 (NT1-RO) if the sampling occurred within 1 year of symptoms onset, and as long-lasting NT1 if the sample was obtained more than 1 year after disease onset. The control group consisted of subjects (*n* = 32) who had been hospitalized for suspected central hypersomnia, which was not confirmed by normal neurophysiological evaluations, including actigraphy, 48-hour polysomnography, and multiple sleep latency tests (MSLT) with documented sleep onset REM periods (SOREMPs).^9^ All control subjects had normal HCRT-1 levels (>200 pg/mL) in their CSF. Exclusion criteria included the presence of other concomitant neurological conditions, psychiatric disorders, acute allergies, recent infections or immunologic diseases, and the use of steroids, anti-inflammatory or immunosuppressive drugs. The local ethics committee (Comitato Etico Interaziendale Bologna-Imola, CE-BI, Prot. Num. 17009) approved the study, and all participants provided written informed consent.

### Collection of CSF and peripheral blood

CSF samples were consecutively collected under fasting conditions from both NT1 patients and controls for HCRT-1 measurement. The first aliquot (1 mL) of CSF was analysed using routine laboratory testing procedures to exclude blood contamination. The remaining CSF was centrifuged at 300 g for 10 min at 4°C, and the supernatant was aliquoted and frozen for HCRT-1 measurement, circulating cell-free DNA extraction and cytokine analysis. Peripheral blood was collected simultaneously with the lumbar puncture into a 10 mL EDTA tube and centrifuged (15 min at 2900 g) to isolate the buffy coat.

### Total DNA and circulating cell-free DNA isolation

Total DNA was isolated from the buffy coat using a Blood DNA Purification System kit on a Maxwell 16 automated instrument (Promega Corporation, Madison, WI, USA). Circulating cell-free DNA was extracted from 400 μL of CSF samples using the MagMAX™ Cell-free DNA Isolation Kit (A29319, Thermo Fisher, Waltham, MA, USA), following the manufacturer’s instructions. Finally, circulating cell-free DNA was eluted in 20 µL of ultrapure water.

### HLA typing and HCRT-1 measurement

The presence of the HLA DQB1*06:02 allele was analysed in all participants by multiplex PCR, as previously described.^10^ HCRT-1 levels were determined in duplicate for each CSF sample using a standard validated direct I125 radioimmunoassay (Phoenix Pharmaceuticals, Belmont, CA, USA).^11^

### Assessment of mtDNA and ccf-mtDNA

Ccf-mtDNA was analysed using droplet digital PCR (QX200™ Droplet Digital™ PCR System, BIO-RAD, ddPCR) with multiplex probe-based method, as previously described.^6^ Briefly, for ccf-mtDNA quantification, we amplified the *MT-ND2* as mtDNA region and *FASLG* as nuclear DNA region. To evaluate the presence of deleted mtDNA molecules, we amplified *MT-ND1* and the breakpoint junction of the 4977 bp ‘common deletion’ of mtDNA.^12^ Five microliters of template was used for each reaction. Additional details are available in the Supplementary Tables 1 and 2.

Ccf-mtDNA was expressed as copies of the target gene per microliters of template analysed (copies/μL template).

Mitochondrial DNA content was assessed by real-time PCR (LightCycler480®, Roche, Basel, Switzerland) through absolute quantification, with the same multiplex assay used for ccf-mtDNA assessment, as previously described.^13^ MtDNA content was expressed as mtDNA copies/cell. Five total DNA samples were excluded from the quantification of mtDNA copies due to poor sample quality.

### Cytokines determination

For the quantification of inflammatory cytokines, we utilized AlphaLISA assays (AlphaLISA kit, Revvity), in accordance with manufacturer’s instructions. We quantified several cytokines in CSF samples, excluding three or four subjects (depending on the cytokine considered) due to unavailability of their CSF: interferon α (INFα, AL297C, Revvity), tumor necrosis factor α (AL3157C, Revvity), interleukin-6 (IL-6, AL223C, Revvity), interleukin-1β (IL1β, AL3160C, Revvity), and interleukin-18 (IL-18, AL3137C, Revvity). The analyses were conducted using AlphaLISA™ technology (Revvity, Waltham, MA, USA).

### Statistical analysis

Statistical analyses were performed using GraphPad Prism 10.0 and R (version 4.3.3) software. Comparisons between control and NT1 groups were assessed using Mann–Whitney U-test. The normality of data distribution was verified using the Kolmogorov–Smirnov and Shapiro–Wilk tests. Differences between ccf-mtDNA on the basis of NT1 onset were analysed using Kruskal–Wallis test followed by the Benjamini and Hochberg *post hoc* test.

For cytokine assessments, outliers in the control group were identified using the ROUT method (Q = 1%)^14^ and excluded from the analysis. Comparisons between the control and NT1 groups were assessed using unpaired *t*-test with Welch’s correction or Mann–Whitney U-test accordingly to data distribution. Spearman’s correlation coefficients were computed to measure the degree of association between HCRT-1 and ccf-mtDNA levels. Univariable linear regression models were used to test the linear relationship between ccf-mtDNA and age/body mass index. All results were considered statistically significant at *P* ≤ 0.05. Finally, we tested the effect size (unpaired mean difference) of the SOREMPs at MSLT test on ccf-mtDNA levels by computing the Cumming estimation plot.

## Results

### Clinical and demographic characteristics of patient and control groups

Clinical and demographic characteristics of the subjects studied are presented in Table 1.

**Table 1:** Clinical and demographic characteristics of NT1 patients and controls

Variable	Narcolepsy type 1 (*n* = 46)	Controls (*n* = 32)
Sex, male	31 (67%)	18 (56%)
Age	23 (±16.98)	24 (±13.60)
BMI	26 (±6.71)	22 (±4.77)
HLA DQB1*06:02	46 (100%)	14 (44%)
CSF HCRT-1 (pg/mL)	20 (±32.89)	341 (±42.74)
SOREMPs	4 (±1.12)	0 (±0.36)
Sleep medications	19	0

Data are represented with ± standard deviation or percentage. BMI, body mass index; HCRT, hypocretin-1; SOREMP, sleep onset REM period during multiple sleep latency test.

All NT1 patients had the HLA DQB1*06:02 allele, CSF HCRT-1 deficiency, pathological SOREMPs at MSLT test and cataplexy. In the control group, 14 subjects were positive for HLA DQB1*06:02 allele. The NT1 cohort was divided into two groups based on disease duration: NT1-RO, which included 18 subjects, and long-lasting NT1, which comprised 28 subjects. Nineteen NT1 patients were under sleep medications administration (Modafinil, Sodium Oxybate or Venlafaxine). Finally, there were no significant differences in gender or age between the NT1 and control groups. However, there was a significant increase in body mass index among NT1 patients (*P* = 0.004), consistent with previous reports.^15^

### Ccf-mtDNA is elevated in CSF of NT1 patients and negatively correlates with HCRT-1 levels

**Figure 1:**
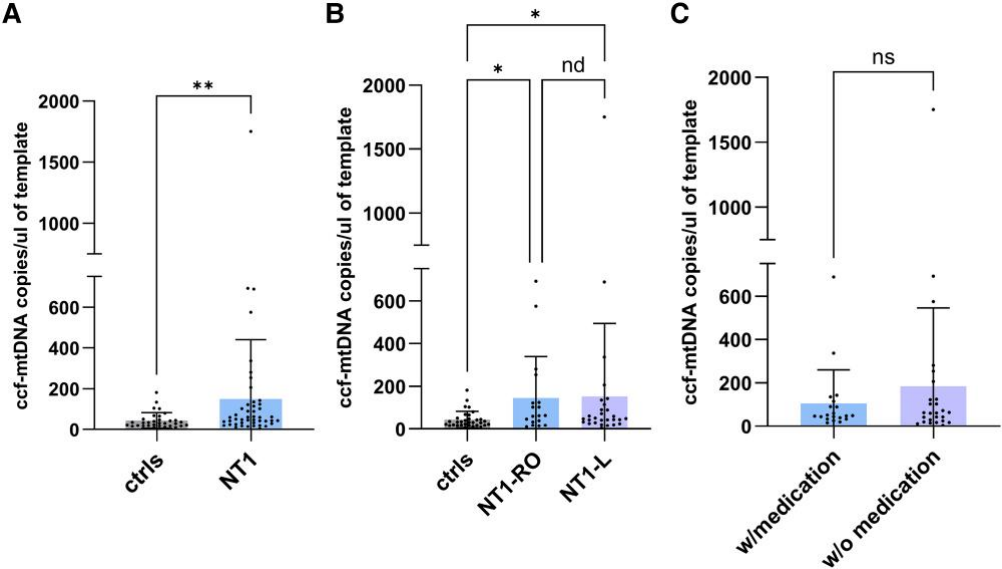
**Levels of ccf-mtDNA in the cerebrospinal fluid of narcolepsy type 1 patients**. (**A**) Plot showing ccf-mtDNA levels in the CSF of NT1 patients (*N* = 46) and control subjects (*N* = 32). Normality test was performed (Shapiro–Wilk test, *P* < 0.0001) and nonparametric analysis was applied using the Mann-Whitney U*-*test (*P* = 0.005). (**B**) A data plot of ccf-mtDNA levels subgrouped by NT1-RO (*N* = 18) and NT1-L (*N* = 28) compared with the control group (*N* = 32). Normality distribution was assessed using the Shapiro–Wilk test (*P* < 0.0001), and the data were analysed using the Kruskal–Wallis test (*P* = 0.02), with correction applied using the Benjamini–Hochberg method. (**C**) Mann–Whitney U*-*test comparing ccf-mtDNA levels between NT1 drug-naïve patients (*N* = 26) and NT1 patients under sleep medications *N* = 20, *P* = 0.88. A nonparametric test was applied after confirming non-normal distribution using the Shapiro–Wilk test (*P* < 0.0001). Each data point represents one single subject analysed. ccf-mtDNA, circulating cell-free mtDNA; NT1, narcolepsy type 1, NT1-RO, NT1 recent disease onset; NT1-L, NT1 late disease onset, with sleep medications (w/medication), without sleep medications (w/o medication).

We evaluated mtDNA release (ccf-mtDNA*, MT-ND2* region) in the CSF of patients with NT1. We found a 3.4-fold increase in NT1 patients compared with control subjects [mean of 150.5 ± standard deviation (SD) 290.0 ccf-mtDNA copies/μL in NT1 versus mean of 44.78 ± SD 39.10 ccf-mtDNA copies/μL in controls; *P* = 0.005, Fig. 1A]. Amplification of a different mtDNA region (*MT-ND1*) showed comparable results (see Supplementary Fig. 1). Moreover, we excluded the presence of deleted ccf-mtDNA molecules in both controls and NT1 patients through the quantification of the breakpoints junction of the 4977 common deletion in the mtDNA. We also quantified the ccf-nuclear DNA in CSF, without highlighting any differences in its levels between NT1 patients and controls (see Supplementary Fig. 1).

Next, we failed to observe any differences in ccf-mtDNA levels based on the disease duration. Both NT1-RO (mean of 145.8 ± SD 194.0) and long-lasting NT1 (mean of 153.5 ± SD 341.2) showed higher levels of ccf-mtDNA compared with controls, with no significant difference between the two groups (Fig. 1B). Additionally, we found no differences in ccf-mtDNA levels between drug-naïve patients and NT1 subjects taking sleep medications (Fig. 1C), with means of 184.8 ± SD 361.0 and, 105.8 ± SD 154.8, respectively.

**Figure 2:**
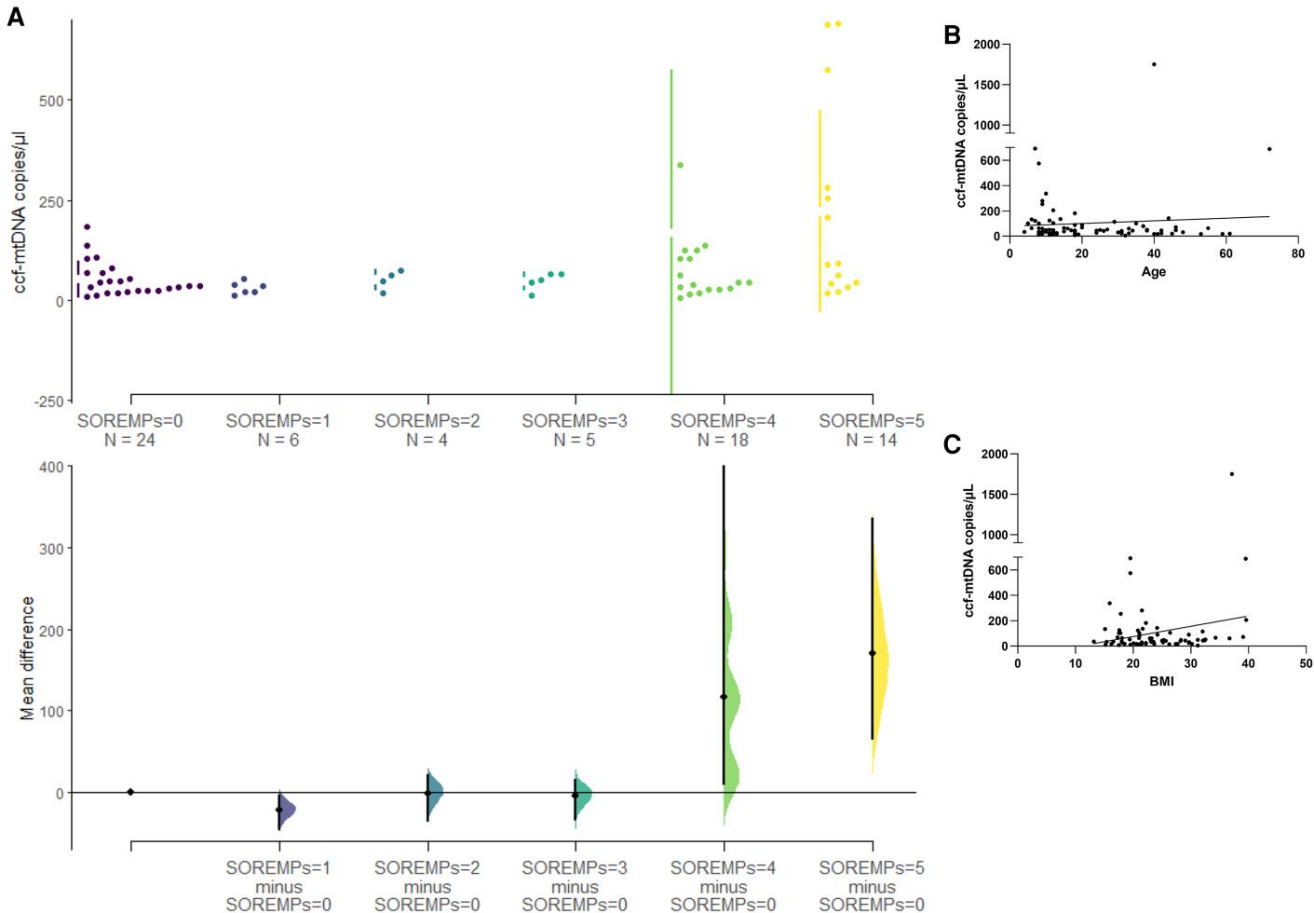
**Relation between ccf-mtDNA levels and clinical parameters in the studied population.** (**A**) Cumming estimation plot between ccf-mtDNA and SOREMP (*N* = 71, SOREMP data was not available for seven subjects) showing the bootstrap effect sizes below the raw data, and also displaying the mean (gap) and ± standard deviation of each group (vertical ends) as gapped lines. Specifically, a ‘shared control’ experimental paradigm was used, where several test samples are compared against a common reference one (SOREMPs =0). The estimated effect size was the mean difference (Δ), which is indicated by the black circle, while 95% confidence interval of Δ is illustrated by the black vertical line. The curve indicates the resampled distribution of Δ, given the observed data. Each plot appears to display: (i) a swarm plot (top): unlike traditional scatter plots, this type of graph arranges data points to prevent overlap, providing a clear view of the distribution and density of individual measurements (ccf-mtDNA levels) across different categories (SOREMP subgroups); (ii) an estimation plot (bottom): this plot uses the *y*-axis to display effect sizes (mean differences) computed via bootstrap resampling. The larger points with error bars represent the mean of each category, with error bars indicating the uncertainty of the estimate (such as 95% confidence intervals). The differences between groups are visualized at the bottom, indicating whether one group's measurement is statistically higher or lower than another's. The range of plausible values for these differences is also shown. If the confidence interval crosses zero, the difference may not be statistically significant; if it does not cross zero, it suggests a meaningful difference between the groups. (**B**) Univariate linear regression model (*F* = 0.42, 95% CI −2.18 to 4.30, *P* = 0.52) with Pearson’s correlations between ccf-mtDNA levels and age (*N* = 78, *r* = 0.07, 95% CI −0.14 to 0.28, *P* = 0.52). (**C**) Univariate linear regression model (*F* = 3.75, 95% CI −0.24 to 16.42, *P* = 0.05) with Pearson’s correlations between ccf-mtDNA levels and BMI (*r* = 0.22, 95% CI −0.006 to 0.42, *P* = 0.05). *N* = 76, BMI data were not available for two subjects. Each data point represents one single subject analysed. BMI, body mass index; circulating ccf-mtDNA, cell-free mtDNA; SOREMP, sleep onset rapid eye movement (REM) periods.

Finally, we observed a moderate negative correlation between ccf-mtDNA and HCRT-1 levels in the CSF (*r* = −0.35, 95% CI −0.54 to −0.13, *P* = 0.001). Also, the ccf-mtDNA mean difference between SOREMPs = 5 and SOREMPs = 0 at the MSLT was 169.5 (95% CI 65.8 to 335.3, Fig. 2A). Furthermore, we did not observe any linear relation between CSF ccf-mtDNA and age (*r* = 0.07, 95% CI −0.14 to 0.28, *P* = 0.52, Fig. 2B), whereas a tendency close to significance emerged with body mass index (*r* = 0.22, 95% CI −0.006 to 0.42, *P* = 0.05, Fig. 2C).

### CSF cytokines analysis revealed decreased IL-6 and increased IL-18 in NT1 patients

**Figure 3:**
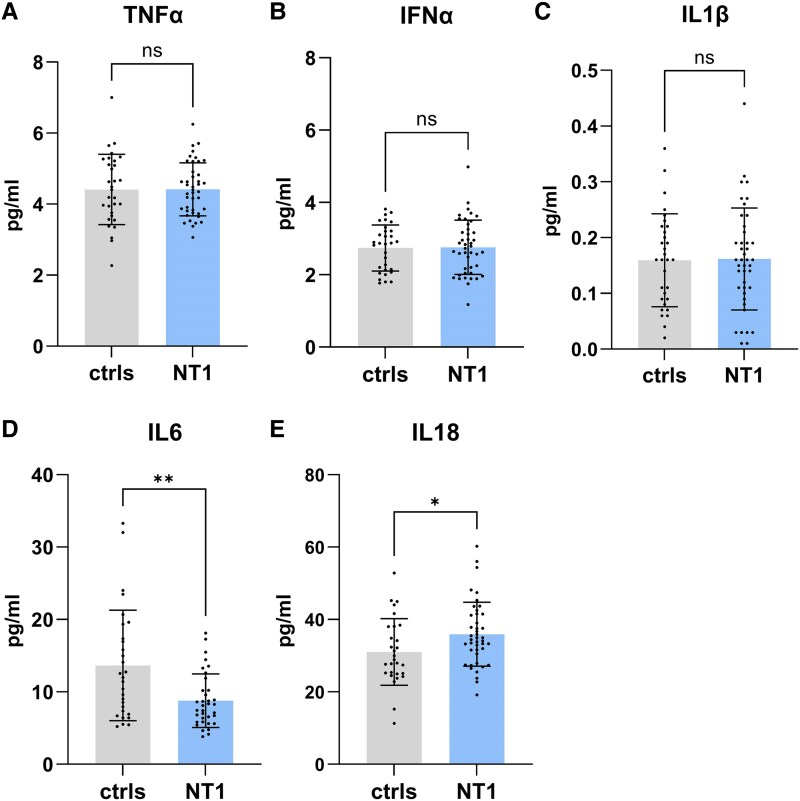
**Cytokine levels in the CSF of narcolepsy type 1 patients compared with control groups.** TNFα levels in CSF of NT1 (*N* = 42) and control (*N* = 31) groups (*P* = 0.98). INFα levels in CSF of NT1 (*N* = 43) and control (*N* = 31) groups (*P* = 0.92). IL-1ß levels in CSF of NT1 (*N* = 42) and control (*N* = 31) groups (*P* = 0.91). IL-6 levels in CSF of NT1 (*N* = 35) and control (*N* = 29) groups (*P* = 0.006). IL-18 levels in CSF of NT1 (*N* = 43) and control (*N* = 29) groups (*P* = 0.03). We applied Welch’s *t*-test for the analysis of TNFα, IFNα, IL-1β, and IL-18 data analysis after verifying normal distribution using the Kolmogorov–Smirnov test (*P* > 0.1000). In contrast, IL-6 exhibited a non-normal distribution (Shapiro–Wilk test, *P* < 0.006), and thus, the Mann–Whitney U-test was performed. Cytokine assays were not performed in three or four subjects (depending of the considered cytokine) due to the unavailability of CSF. Each data point represents one single subject analyzed. INFα, interferon α; IL, interleukin; NT1, narcolepsy type 1; TNFα, tumor necrosis factor α.

Cytokine analysis in NT1 patients revealed an imbalance in certain interleukins (Fig. 3). Specifically, we observed a decreased level of IL-6 in patients compared with controls (*P* = 0.007), as well as an increased level of IL-18 (*P* = 0.02, Fig. 3D and E, respectively). We did not observe significant differences in the levels of other cytokines, such as tumor necrosis factor α, INFα, and IL-1β (Fig. 3A, B and C).

### Blood mtDNA copy number was not altered in NT1

**Figure 4:**
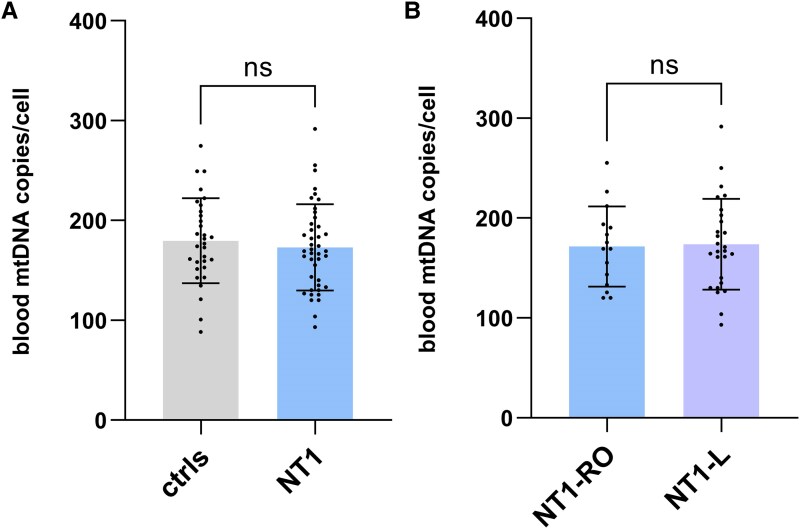
**Mitochondrial DNA copy number evaluated in the blood.** Unpaired *t*-tests were used for the analyses after assessing the normal distribution of data (Kolmogorov–Smirnov test, *P* > 0.1000). Blood DNA was not available for five subjects. Each data point represents one single subject analyzed. (**A**) mtDNA copy number in the peripheral blood of NT1 patients (*N* = 42) compared with controls (*N* = 31, *P* = 0.52). (**B**) Comparison of blood mtDNA copy number between NT1-RO (*N* = 15) and NT1-L (*N* = 27) patients (*P* = 0.87). NT1, narcolepsy type 1; NT1-L, NT1 long lasting disease; NT1-RO, NT1 recent disease onset.

We quantified the mtDNA content in blood cells of NT1 patients. We found no statistically significant differences in peripheral mtDNA copies between the NT1 and control groups (Fig. 4A). We also analysed the mtDNA content subdividing NT1 patients based on their disease onset, but also in this case we failed to observe any differences (Fig. 4B).

## Discussion

To the best of our knowledge, this is the first study assessing ccf-mtDNA levels in the CSF of NT1 patients. We showed an increased amount of ccf-mtDNA in NT1 patients compared with the control group. Moreover, patients with the highest number of SOREMPs at the MSLT exhibited elevated levels of ccf-mtDNA, which were negatively correlated with CSF HCRT-1, raising the issue of mitochondrial involvement in the pathophysiology of NT1. Furthermore, we observed concomitant alterations in cytokine concentrations in the CNS of NT1 patients, supporting the presence of inflammation. Finally, age did not impact ccf-mtDNA levels and a tendency close to significance was found in relation to body mass index.

Ccf-mtDNA acts as a damage-associated molecular pattern by activating specific inflammatory pathways and promoting a sterile immune response.^5^ Since mitochondria have a bacterial origin, mtDNA shares similarities with bacterial DNA; therefore, once released from mitochondria, it is recognized by pattern recognition receptors as foreign DNA, activating pathways involving the interferon response, toll-like receptor 9, and the inflammasome (NLRP3). These pathways trigger inflammatory and immune responses through the release of pro-inflammatory cytokines.^5^ Moreover, ccf-mtDNA release may result from cellular stress associated with mitochondrial dysfunction or cellular injury.^16^

Only a few studies assessed ccf-mtDNA in CSF of pathological conditions. For example, a recent study^17^ examined CSF ccf-mtDNA in patients with idiopathic REM sleep behaviour disorder finding higher amounts of deleted ccf-mtDNA in these patients compared with controls. The authors concluded that high amounts of deleted ccf-mtDNA were related to the primary mitochondrial pathophysiological mechanism believed to precede the clinical manifestation of Lewy body disease.^17^ In our study, the ccf-mtDNA levels did not discriminate NT1 patients with recent onset, close to the active hypocretinergic neuronal loss, from those more chronic. As ccf-mtDNA negatively correlated with the reduction of CSF HCRT-1 levels, our working hypothesis is that mitochondrial alterations may persist over time after the onset of clinical symptoms as a consequence of the loss of hypocretinergic neurons. We also failed to detect deleted mtDNA as judged by assessing the breakpoints junction of the so-called ‘common’ deletion.

HCRTs are excitatory neuropeptides produced by neurons exclusively located in lateral and posterior hypothalamic regions, which widely project to all areas of the brain.^18^ Brain regions reached by HCRT are important for arousal and help controlling the response to stress.^18^

In this context, HCRT-1 is also implicated in regulating genes involved in mitochondrial biogenesis and dynamics. Multiple *in vitro* studies^19,20^ showed that HCRT-1 increased mitochondrial membrane potential and resulted in increased ATP production and that HCRT-1 exerts neuroprotection by reducing inflammation, promoting neuronal survival, and protecting cells from death caused by oxidative and hypoxic stress. Conversely, numerous *in vivo* and *in vitro* studies^3^ have shown that HCRT-1 deficiency is linked to neurodegeneration, memory and cognitive deficits and neuroinflammation, affecting CNS. In a rat model of cerebral ischaemia, hcrt-1 exerts its effects by attenuating apoptosis, decreasing astrocyte activation, and reducing pro-inflammatory cytokine production through the inhibition of several molecular signalling pathways.^21^ Moreover, HCRT-1 enhances the mammalian target of rapamycin activity resulting in increased cell proliferation.^22^ Mammalian target of rapamycin also plays a role in the direct or indirect inhibition of autophagy. During autophagy, macromolecules and dysfunctional components of the cell are removed by lysosomal-dependent mechanisms.^22^ Thus, all considered, we hypothesize that lack of HCRT-1 in the CNS of NT1 patients may lead to reduced activation of mammalian target of rapamycin, causing excessive autophagy that disrupts mitochondrial homeostasis, potentially increasing apoptosis and inflammation. Moreover, sleep deprivation disrupts neuronal networks and induces excessive autophagy, as evidenced by an increase in autophagosomes and exacerbated apoptosis.^23,24^ While autophagy is crucial for cellular homeostasis, its excessive activation beyond lysosome degradation capacity can ultimately trigger apoptosis and consequently lead to the release of mtDNA. Taken together, all these mechanisms may be implicated with our finding of higher ccf-mtDNA in NT1. This process appears to be specific to the CNS, as peripheral HCRT-1 levels^25^ and blood mtDNA copy numbers were normal in NT1 patients. Ultimately, neuronal stress in the CNS may lead to mtDNA release and activation of inflammatory response pathways, resulting in cytokine production, as supported by the cytokine imbalance we observed, with decreased IL-6 and increased IL-18 levels.

Of note, cytokines play an important role in maintaining normal sleep, and altered levels have been linked to sleep disorders.^26^ Moreover, cytokine profiles can be modulated by a disrupted sleep–wake cycle, which principally affects IL-6 and tumor necrosis factor α levels. This fit our observation of lower IL-6 levels as already reported in a meta-analysis.^26^ Studies in rodents had already shown that IL-6 impacts the physiological balance between non-REM and REM sleep.^27^ Similarly, in humans, IL-6 has been shown to increase slow-wave activity during slow-wave sleep, a deep sleep stage essential for cerebral restoration and recovery.^28^ NT1 is primarily characterized by excessive daytime sleepiness, but its hallmark feature is abnormal REM sleep regulation.^29^ REM sleep disturbances in NT1 can manifest as cataplexy, sleep paralysis, sleep-related hallucinations, REM sleep behaviour disorder, abnormal dreams, polysomnographic evidence of REM sleep disruption, sleep-onset REM periods and fragmented REM sleep.^29^ Considering these findings, the low IL-6 levels observed in the CSF of NT1 patients may align with the pathophysiological characteristics of the disorder.

Pleiotropic IL-18 is an important modulator of the immune response and is expressed in the CNS, taking part in neuroinflammatory and neurodegenerative processes.^30^ In a murine model of encephalitis,^31^ both IL-18 and IL-1β were produced by microglia and astroglia, exhibiting pro-inflammatory activities. Overall, IL-18 plays an important role in regulating innate immunity and autoimmune diseases and can be activated by ccf-mtDNA, which promotes the assembly of NLRP3^32^ and the pyroptosis signalling cascade.

Currently, it remains unexplored if release of ccf-mtDNA is an event downstream the onset of NT1 mainly related to decreased HCRT-1 level or mitochondrial dysfunction may be also implicated in the primary pathogenesis of NT1. Remarkably, it is even questioned if hypocretinergic neurons are lost by a sudden wave of cell death due to an autoimmune process affecting very specifically this neuronal subpopulation or these neurons are simply silenced by epigenetic mechanisms as recently proposed.^33^

This study has several limitations. First, the sample size was relatively small, which is common in studies involving rare diseases like NT1. A larger cohort would provide more robust data possibly strengthening our current conclusions. While we observed significant alterations in ccf-mtDNA and cytokine levels, functional studies using *in vitro* models, such as neuronal cells, may shed light on how to interpret the current data, clarifying how HCRT-1 and cytokines may affect mitochondrial function and neuronal viability in NT1 disease.

In conclusion, our findings represent the first evidence of elevated levels of ccf-mtDNA in NT1, highlighting the complex interplay between mitochondrial dysfunction, neuroinflammation, and cytokine alterations in this sleep disorder. More studies are needed to unravel the underlying mechanisms and their possible implications in terms of therapeutic interventions.

## Supplementary Material

fcaf125_Supplementary_Data

## Data Availability

The data supporting the findings of this study are available on Zenodo (accession number: 10.5281/zenodo.14959703).
