# Supplementary material for: Elevated circulating cell-free mitochondrial DNA level in cerebrospinal fluid of narcolepsy type 1
Source: Brain Commun. 2025 Apr 17;7(2):fcaf125. doi: 10.1093/braincomms/fcaf125 (PMC12003949; doi:10.1093/braincomms/fcaf125)
Supplement: fcaf125_Supplementary_Data [file fcaf125_supplementary_data.docx]

Elevated circulating cell-free mitochondrial DNA level in cerebrospinal fluid of narcolepsy type 1

Monica Moresco^1^, Concetta Valentina Tropeano^1^, Martina Romagnoli^1^, Giulia Neccia^1^, Alessandro Rapone^2^, Fabio Pizza^1,2^, Stefano Vandi^1,2^, Emmanuel Mignot^3^, Alessandra Maresca^1*^, Valerio Carelli^1,2,†^, Giuseppe Plazzi^1,4,†^

^†^Valerio Carelli and Giuseppe Plazzi contributed equally to this work.

Author affiliations:

1. IRCCS Istituto delle Scienze Neurologiche di Bologna, Bologna, 40139, Italy
2. Department of Biomedical and Neuromotor Sciences, University of Bologna, Bologna, 40126, Italy
3. Stanford Center for Sleep Sciences and Medicine, Stanford University School of Medicine, Stanford, CA 94305, USA
4. Department of Biomedical, Metabolic and Neural Sciences, University of Modena and Reggio Emilia, Modena, 42121, Italy

*Correspondence to: Alessandra Maresca

IRCCS Istituto delle Scienze Neurologiche di Bologna, Programs of Neurogenetics, Ospedale Bellaria, Via Altura n 3, 40139, Bologna, Italy

E-mail: alessandra.maresca@isnb.it

**Supplementary Materials**

**Materials and methods:**

**Assessment of mtDNA and circulating cell free-mtDNA**

Regarding circulating cell free-mtDNA (ccf-mtDNA) quantification we performed multiplex ddPCR assay allowing simultaneous detection of mitochondrial *MT-ND2* region and nuclear DNA (nDNA). The reaction mix was prepared in 22 µL final volume containing 5 µL of previously extracted ccf-DNA. We used previously published probes and primers for *MT-ND2* and *FASLG*^1^ showed in Supplementary Table 1.

| *MT-ND2* Forward | 5′-CACAGAAGCTGCCATCAAGTA-3′ |
| --- | --- |
| *MT-ND2* Reverse | 5′-CCGGAGAGTATATTGTTGAAGAG-3′ |
| *MT-ND2* Probe | 5′-[FAM]-CCTCACGCAAGCAACCGCATCC-[BHQ1]-3′ |
| *FASLG* Forward | 5′-GGCTCTGTGAGGGATATAAAGACA-3′ |
| *FASLG* Reverse | 5′-AAACCACCCGAGCAACTAATCT-3′ |
| *FASLG* Probe | 5′[HEX]-CTGTTCCGTTTCCTGCCGGTGC-[BHQ1]-3′ |

**Supplementary Table 1**. Primers and probes used in multiplex ddPCR assay for detection of mitochondrial (*MT-ND2*) and nuclear DNA (*FASLG*).

We performed an additional multiplex ddPCR amplifying *MT-ND1* and the breakpoint junction of the 4977 bp common deletion of mtDNA. We used probes and primers showed in Supplementary Table 2.

| *MT-ND1* Forward | 5′-CCCTAAAACCCGCCACATCT-3′ |
| --- | --- |
| *MT-ND1* Reverse | 5′-GAGCGATGGTGAGAGCTAAGGT-3′ |
| *MT-ND1* Probe | 5′-[FAM]-CCATCACCCTCTACAACCGCCC-[BHQ1]-3′ |
| *Common deletion region* Forward | 5′-CCTTACACTATTCCTCATCACC-3′ |
| *Common deletion region* Reverse | 5′-TGTGGTCTTTGGAGTAGAAACC-3′ |
| *Common deletion region* Probe | 5′[HEX]-TGGCAGCCTAGCATTAGCAGG-[BHQ1]-3′ |

**Supplementary Table 2**. Primers and probes used in multiplex ddPCR assay for detection of mitochondrial *MT-ND1* and the breakpoint junction of the 4977 bp common deletion region of mitochondrial DNA.

**Results:**

**Ccf-nDNA is not altered in NT1**

As detailed in Materials and Methods section, we simultaneously analyzed the ccf-mtDNA (*MT-ND2)* and the ccf-nDNA (*FASLG*) levels in NT1 patients compared to controls. Regarding ccf-nDNA we did not observe any statistically significant difference between controls and NT1 patients, as shown in Supplementary Figure 1 A.

**Ccf-mtDNA *MT-ND1* is elevated, similar to ccf-mtDNA *MT-ND2*, in the CSF of NT1 patients**

Amplification of a different mtDNA region (*MT-ND1*) showed comparable results to *MT-ND2*, supporting the increased level of ccf-mtDNA in the CSF of NT1 patients, as shown in Supplementary Figure 1 B. We then proceeded to confirm the relationship between ccf-mtDNA copies of these two different regions (*r*= 0.890, 95% CI 0.82 to 0.93, p<0.0001, Supplementary Figure 1 C).

**References:**

1. Mussini C, Pinti M, Bugarini R, Borghi V, Nasi M, et al. Effect of treatment interruption monitored by CD4 cell count on mitochondrial DNA content in HIV-infected patients: a prospective study. AIDS. 2005 ;19:1627-33.
2. Pyle A, Brennan R, Kurzawa-Akanbi M, Yarnall A, Thouin A, Mollenhauer B, Burn D, Chinnery PF, Hudson G. Reduced cerebrospinal fluid mitochondrial DNA is a biomarker for early-stage Parkinson's disease. Ann Neurol. 2015 Dec;78(6):1000-4. doi: 10.1002/ana.24515. Epub 2015 Nov 13. PMID: 26343811; PMCID: PMC6551217.


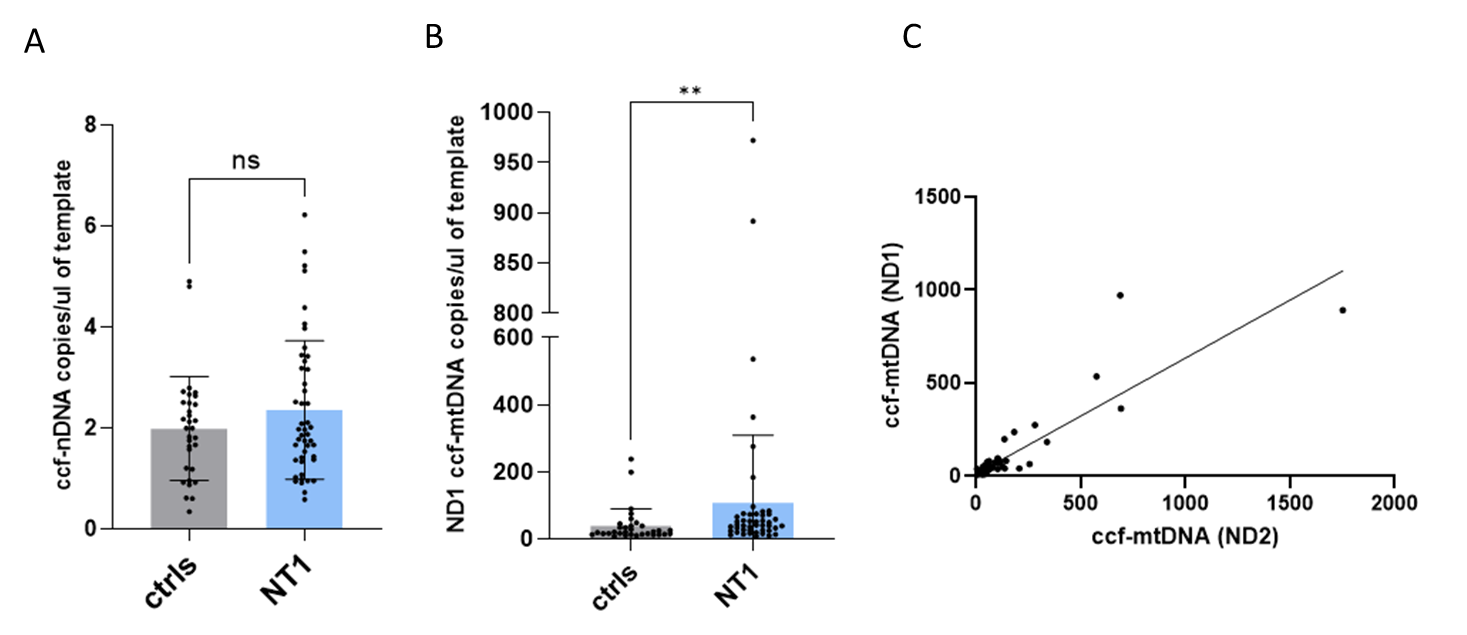


**Supplementary Figure 1. Ccf- mtDNA and ccf-nDNA evaluation in CSF from controls and NT1 patients.**

1. The data plot showed ccf-nDNA levels in CSF of NT1 patients (N=46) compared to controls (N=32). Normality test was performed (Shapiro-Wilk test, p < 0.007) and nonparametric analysis was applied using the Mann-Whitney *U-*test (p = 0.444).
2. The data plot showed ccf-mtDNA (*MT-ND1*) level in CSF of NT1 patients (N=46) compared to controls (N=32). Normality test was performed (Shapiro-Wilk test, p <0,0001) and nonparametric analysis was applied using the Mann-Whitney *U-*test (p = 0.001).
3. Pearson’s correlation between *MT-ND2* and *MT-ND1* assays for ccf-mtDNA (N=78) in the CSF (*r*=-0.89, 95% CI 0.83 to 0.93, p <0,0001).

Each data point represent one single subject analysed.

Abbreviations: circulating cell-free mtDNA (ccf-mtDNA), nuclear DNA (nDNA), narcolepsy type 1 (NT1).
